# Characteristics of the copper‐induced viable‐but‐non‐culturable state in bacteria

**DOI:** 10.1007/s11274-021-03006-5

**Published:** 2021-02-05

**Authors:** Laurens Maertens, Jean-Yves Matroule, Rob Van Houdt

**Affiliations:** 1 Microbiology Unit, Interdisciplinary Biosciences, Belgian Nuclear Research Centre (SCK CEN), Mol, Belgium; 2 Research Unit in Microorganisms Biology (URBM), Narilis Institute, University of Namur, Namur, Belgium

**Keywords:** Copper, Viable‐but‐non‐culturable, Oxidative stress, Oligotrophic

## Abstract

The antimicrobial applications of copper (Cu) are exploited in several industries, such as agriculture and healthcare settings. While Cu is capable of efficiently killing microorganisms, sub-lethal doses can induce a viable-but-non-culturable (VBNC) state in bacteria of many distinct clades. VBNC cells cannot be detected by standard culture-based detection methods, and can become a threat to plants and animals as they often retain virulent traits upon resuscitation. Here we discuss the putative mechanisms of the Cu-induced VBNC state. Common observations in Cu-induced VBNC cells include a cellular response to reactive oxygen species, the exhaustion of energy reserves, and a reconfiguration of the proteome. While showing partial overlap with other VBNC state-inducing stressors, these changes seem to be part of an adaptive response to Cu toxicity. Furthermore, we argue that Cu resistance mechanisms such as P-type ATPases and multicopper oxidases may ward off entry into the VBNC state to some extent. The spread of these mechanisms across multi-species populations could increase population-level resistance to Cu antimicrobials. As Cu resistance mechanisms are often co-selected with antibiotic resistance mechanisms, this threat is exacerbated.

## Introduction

The antimicrobial properties of copper (Cu) have seen renewed interest in recent decades, in part due to the widespread increase in bacterial resistance to organic antibiotics. Cu-based antimicrobials are widely applied in the agriculture industry, e.g. to combat foot-rot in cattle and sheep (Rensing et al. 2018), and downy mildew in viticulture (Komarek et al. 2010). In addition, Cu surfaces can be an integrated part of decontamination strategies in the food industry, since they effectively kill common bacterial pathogens such as *Salmonella enterica* and *Campylobacter jejuni* (Parra et al. 2018; Faúndez et al. 2004). However, the most promising applications of antimicrobial copper are found in the medical field, where antimicrobial-resistant (AMR) (opportunistic) pathogens pose severe and increasing risks to patients and personnel (Cassini et al. 2019). For instance, the prevalence of healthcare-associated infections (HAIs), often by AMR strains, has been linked to microbial contamination of high-touch surfaces (Otter et al. 2013; Boyce 2007, 2016).

Cu antimicrobials come in several distinct formulations, often tailored to the specific application. For instance, Cu salts and organic complexes are used as fungicides (Wang et al. 2018) and algicides (Shen et al. 2019), and Cu-doped zeolites are being investigated to simultaneously disinfect and remove metals from wastewater (Fanta et al. 2019). Cu salt-based formulations can be applied topically to combat infections, e.g. herpes (Clewell et al. 2012; Chen et al. 2016) and vaginitis (Abbott and Abbott 2014). In addition, several Cu salt-based therapies have been developed in recent years (Clewell et al. 2012; Styczynski et al. 2015). In parallel, a surging contribution of Cu nanoparticle (CuNP) formulations, which are intrinsically of interest due to their high surface to volume ratio, has been noted, again often in healthcare settings. For applications, CuNPs can be incorporated into polymeric matrices such as textiles, glass, and polypropylene, as reviewed by Tamayo et al. (2016) and Borkow (2014). These CuNP-infused matrices convey several key advantages, among which the controlled release of Cu ions and the increased surface area of the antimicrobial material. Such materials can be used for food packaging, water disinfection and medical applications. For example, CuNP-containing cotton and bamboo rayon clothing is toxic towards *Staphylococcus aureus*, which is associated with many HAIs (Anita et al. 2011; Perelshtein et al. 2009; Teli and Sheikh 2013). A final formulation of Cu antimicrobials comes in the shape of solid surfaces of Cu metal and its alloys. Noyce et al. (2006) found that a positive correlation exists between the killing rates of Cu alloy surfaces and their Cu content. The potential antimicrobial applications of Cu surfaces have been reviewed by Vincent et al. (2016), who emphasized their utility in hospital environments owing to their ability to efficiently inactivate HAI-associated strains such as methicillin-resistant *S. aureus* and vancomycin-resistant enterococci.

## Mechanisms of copper toxicity

While highly toxic at excess amounts, Cu ions function as a micronutrient and intracellular Cu ion concentrations must consequently be carefully regulated to achieve viable homeostasis. Specifically related to bacteria, Cu ion toxicity has been shown to have multiple modes of action, and has been expertly reviewed both by Lemire et al. (2013) and Giachino and Waldron (2020). The cell envelope represents an important target since Cu can inhibit the correct enzymatic maturation of lipoproteins by binding to catalytic cysteine residues. This leads to an increased level of toxic intermediates in the inner membrane, in turn activating the envelope stress response (May et al. 2019). In a similar manner, Cu can impede the crosslinking of peptidoglycan and its binding to membrane lipoproteins, thus weakening the cell envelope (Peters et al. 2018). Cu can also oxidize thiol residues involved in the maturation of periplasmic polypeptides, again leading to the buildup of misfolded intermediates (Ito and Inaba 2008).

For CuNPs and Cu surfaces, the direct contact with bacterial cells seems to be an essential facet of toxicity, next to the release of Cu ions (Vincent et al. 2018). This phenomenon is termed ‘contact killing’, and has been elegantly demonstrated by Mathews et al. (2013), by comparing killing rates of naked Cu surfaces to Cu surfaces covered with an inert polymeric grid, inhibiting contact killing but not the release of ions into the medium. Membrane lipid peroxidation has been proposed as a key process in contact killing (Hong et al. 2012; Grass et al. 2011), but the exact mechanism of toxicity remains unclear.

Finally, another general mechanism of Cu toxicity is the generation of reactive oxygen species (ROS), such as hydroxyl and hydroperoxyl radicals, which can damage cellular components. Since it remains difficult to directly identify intracellular ROS, evidence for this toxicity mechanism has been mostly circumstantial. Consequently, the exact contribution of ROS generation to overall Cu toxicity is ambiguous. However, Macomber et al. (2007) have shown that Cu catalyzes hydroxyl radical formation in the periplasm *in vivo*, and Cu-catalyzed ROS generation via a Fenton-like process has been demonstrated in vitro (Valko et al. 2005; Stohs and Bagchi 1995). In addition, the high affinity of the cuprous ion for thiol groups can lead to the depletion of antioxidants like glutathione and the destruction of redox-active Fe-S clusters (Macomber and Imlay 2009; Arguello et al. 2013).

## Copper toxicity induces the VBNC state

It is clear that elevated Cu concentrations are highly toxic to bacteria. In order to understand the bacterial response to this toxicity, it is relevant to study the effects of sub-lethal Cu concentrations. A curious observation in this regard is the apparent loss of culturability of Cu-exposed bacterial cells. At the same time, these cells often retain characteristics of viability such as intact cell membranes and metabolic activity. An overview of tested strains exhibiting this behavior is provided in Table 1. The latter includes human as well as plant pathogens, reflecting the medical and agricultural use of Cu antimicrobials.

**Table 1:** Overview of bacterial species with Cu-induced VBNC state

Bacteria		Induction conditions			Maximal induction efficiency	VBNC state characteristics	Resuscitation conditions	Reference
Species	Strains	Medium	CFU/mL	[Cu^2+^] µM				
*E. coli*	ES80; K-12 (C600), O157:O7 (EDL933), O104:H4 (RKI 01-09591)	S	10^6^ – 10^8^	500	No CFU in 7 days; no CFU in 5 days	IM; IM, decreased cell size	Washing with EDTA, resuspending in saline solution; identical	Grey and Steck (2001a), Aurass et al. (2011)
*P. aeruginosa*	PA14; AdS, DSM 50,071, PAO1	PBS; H_2_O	10^6^ – 10^7^	4–10	6 log in 2 h; no CFU in 10 h	IM; IM, intact rRNA, not cytotoxic	Adding DDTC to medium; identical	Bedard et al. (2014), Dwidjosiswojo et al. (2011)
*R. solanacearum*	AS108; SL341	S; PBS	10^8^	5–500	3.5 log in 9 days at 5 µM; no CFU in 24 h	IM, KP; intact membrane, strong aggregation, lower PHB content, lower RNA content, various proteome changes, increased H_2_O_2_ content	Attempted but not achieved; spontaneous at low CuSO_4_, not observed at higher concentration	Grey and Steck (2001b), Um et al. (2013)
*C. metallidurans*	CH34, AE104	H_2_O	10^8^	10	4 log in 3 h for CH34, no CFU in 1 h for AE104	IM	Spontaneous for CH34, not observed for AE104	Maertens et al. (2020)
*E. amylovora*	CFBP1430, IVIA1892-1	MM	10^7^	5–50	No CFU in 30 days at 5 µM	IM, CP, reduced pathogenicity, increased cell size and envelope thickness	Addition of equimolar (to Cu^2+^) EDTA, asparagine, citric acid, fresh immature pear juice, King’s Broth	Ordax et al. (2006)
*A. citrulli*	AAC00-1	MM	10^7^	0.5–50	2 log in 10 days at 0.5 µM	IM, not pathogenic	Resuspension in LB broth, cell-free supernatant, AB medium + casein hydrolysate, or washing with EDTA	Kan et al. (2019)
*X. axonopodis*	49b	H_2_O	10^6^	45–225	3 log in 30 min at 45 µM	CP, reduced pathogenicity	Addition of EDTA	del Campo et al. (2009)
*A. tumefaciens*	At493	H_2_O	10^8^	50–5000	No CFU in 1 day	IM, KP	Spontaneous at low CuSO_4_ but not observed at higher concentrations	Alexander et al. (1999)
*R. leguminosarum*	F6	H_2_O	10^8^	5–500	No CFU in 1 day	IM, KP	Spontaneous at lowCuSO_4_ but not observed at higher concentrations	Alexander et al. (1999)
*C. michiganensis*	BT0505	S	10^7^	0.05–50	No CFU in 37 days at 0.05 µM	IM	*In planta*, resuspension in LB	Jiang et al. (2016)

*IM* Intact membrane as per LIVE/DEAD staining, *KP* positive test in Kogure assay, *CP* positive test in respiratory activity assay with CTC

The question arises whether these observations are merely a consequence of sustained cell damage, which we will denote as ‘sublethal injury’, or whether it concerns the active induction of a regulated cellular state as a response to the perceived Cu stress. This cellular state is often denoted as the ‘viable-but-non-culturable’ (VBNC) state. An excellent review on the phenotypic characteristics and the mechanisms of identification has been written by Pinto et al. (2015). VBNC cells cannot be cultured on standard laboratory media, but retain membrane integrity, and low but measurable levels of metabolism and gene expression (Davey 2011; Schottroff et al. 2018). They are formed under harsh conditions like starvation and in the presence of various chemical and physical stressors. The VBNC state displays similar traits to the so-called ‘cellular quiescence’, a noted survival strategy of *Mycobacterium tuberculosis*, but no in-depth comparison of these states has been performed (Rittershaus et al. 2013; Betts et al. 2002). It is often difficult to experimentally verify whether a cell population is sub-lethally injured or in the VBNC state, or both, since many measurable properties are shared by both cell states. One key distinction is the inability of sub-lethally injured cells to grow on selective media, while colony growth would be observed on non-selective media, as claimed by Bogosian and Bourneuf (2001). However, it is abundantly clear that no extant culture medium is entirely non-selective. Thus, this definition of sub-lethal injury seems untenable, especially when comparing species over multiple clades, each with their own optimal growth conditions. Consequently, many researchers do not make the above distinction and term both cell states under the VBNC denomination. Furthermore, the evolutionary conservation of entering a VBNC state in response to a range of stress conditions and the growing body of evidence elucidating its molecular mechanisms indicates that it is a cell-programmed phenomenon. Finally, there exists an ongoing discussion about the similarities between VBNC cells and persister cells. Persister cells are slow-growing, antibiotic-tolerant cells that represent a subpopulation of actively dividing cultures. This phenotypic heterogeneity allows for rapid recolonization of habitats after transient stress conditions (Fisher et al. 2017). The VBNC state shares characteristics with persister cells, such as morphology and resuscitation characteristics. Based on these similarities, Kim et al. (2018) suggested that VBNC cells and persister cells are one and the same. However, this conclusion was refuted by Ayrapetyan et al. (2018). Ultimately, an extensive comparison between the VBNC state and the persister phenotype is outside the scope of this review.

A fraction of the VBNC cell population can often be manipulated in order to make them regain culturability. This process is called resuscitation. The ability to show resuscitation is essential when arguing in favor of a programmed VBNC state, and has become a mainstay of VBNC studies (Bogosian and Bourneuf 2001; Pinto et al. 2015). Multiple resuscitation stimuli have been listed by Pinto et al. (2015), but it is difficult to distill information about putative molecular mechanisms considering the large number of experimental conditions and the phylogenetic distance of the tested strains. A note must be made that resuscitation is often difficult to distinguish from regrowth of the remaining culturable population. Protocols such as the analysis of dilution series exist to minimize the likelihood of mistaking regrowth for resuscitation, but they are not consistently used in practice (Whitesides and Oliver 1997). Consequently, this distinction must be approached with caution.

## Mechanisms behind the cu‐induced VBNC state

Evidently, Cu is not the only stress capable of inducing the VBNC state. Common stressors in this regard include starvation, desiccation, low temperature, pH extremes and oxidative compounds like hypochlorous acid (Zhao et al. 2017; Pinto et al. 2015). It seems unlikely that a direct regulatory pathway exists between the sensing of each separate stressor and the induction of a general VBNC state. More plausible would be VBNC induction triggered by one or more common consequences elicited by these distinct stressors. Therefore, we may derive insights from the interpolation of these mechanisms.

Oxidative stress can induce the VBNC state in different species of bacteria (Cuny et al. 2005; Liao et al. 2020). In addition, Li et al. (2014) suggested that the oxidative stress regulator OxyR is involved in the regulation of VBNC state induction, based on work of Longkumer et al. (2014), Abe et al. (2007) and Wang et al. (2013). Oxidative stress is an important component of many conditions able to induce the VBNC state, such as desiccation (Franca et al. 2007), starvation (McDougald et al. 2002) and exposure to common disinfectants like hypochlorous acid (da Cruz Nizer et al. 2020). As mentioned before, it is also a substantial component of Cu toxicity. Kan et al. (2020) showed increased levels of superoxide dismutase and alkyl hydroperoxide reductase proteins in Cu-induced *Acidovorax citrulli* VBNC cells as well as during the early stages of resuscitation. In addition, an increased H_2_O_2_ content was detected in Cu-induced *Ralstonia solanacearum* VBNC cells (Um et al. 2013). Thus, we conclude that oxidative stress elicited by Cu is an important facet of the Cu-induced VBNC state.

The rapid response required for the detoxification of VBNC state-inducing stressors can put a strain on the cellular energy metabolism. For instance, Cu-exposed cells have a lower ATP content, and a decreased electron transport and dehydrogenase activity (Chen et al. 2019; Feng et al. 2020). In addition, this decreased ATP content has been used to define toxic CuNP levels (Reyes et al. 2016). While metabolic activity is by definition maintained in VBNC cells, it is often diminished. ATP levels are often, but not always, lower in VBNC cells (Bai et al. 2019; Kim et al. 2015; Liao et al. 2020). However, ATP content is not a straightforward indicator of general metabolic activity (Parry and Shain 2011) and ATP content in Cu-induced VBNC cells is still to be evaluated. All the while, many proteins involved in central metabolic processes were downregulated in Cu-induced VBNC *Acidovorax citrulli* cells (Kan et al. 2020). In addition, PHB content, which functions as a cellular energy reserve, is reduced in Cu-induced VBNC *Ralstonia solanacearum* cells (Um et al. 2013). Consequently, the decrease of metabolic activity due to Cu stress appears to be another vital facet of the Cu-induced VBNC state.

Degradation of the proteome can derive both directly from the interaction of proteins with Cu ions, and from the destructive action of ROS. In addition, the proteome needs some measure of reconfiguration to cope with the imposed stresses, by repairing degraded peptides and synthesizing stress defense mechanisms. Proteome adaptations are common in VBNC cells (Ramamurthy et al. 2014; Zhao et al. 2017) and in cells undergoing Cu stress (Monchy et al. 2006; Nandakumar et al. 2011). However, to date we could find only a single study investigating the proteomic composition of Cu-induced VBNC cells. In Kan et al. (2020), 3 of the 5 COG classes with the most changes in VBNC and resuscitating cells correspond to some aspect of proteome reconfiguration (posttranslational modification, protein turnover, and chaperones; amino acid and transport; translation, ribosomal structure, and biogenesis). It remains unclear to what extent these changes are due to direct changes as a result of Cu toxicity, or to the adaptive response of the cell to this toxicity. Evidence for the latter was found in the upregulation of a Cu-translocating ATPase and superoxide dismutase enzyme.

Overall, it remains clear that more research is needed to characterize Cu-induced VBNC cells. Additional whole-cell proteome, transcriptome, and metabolome studies would provide valuable insights into the complex but similar behavior exhibited by these phenotypically distinct cells. Likewise, it would be interesting to study the heterogeneity of this behavior via single-cell techniques.

### Copper resistance mechanisms and their role in entry into the VBNC state

We recently showed that the activity of Cu resistance mechanisms (CRMs) affects entering the VBNC state upon exposure to sub-lethal Cu concentrations (Maertens et al. 2020). It was shown that the extent of VBNC induction was markedly decreased in *Cupriavidus* strains containing the pMOL30 megaplasmid, encoding many genes involved in Cu resistance. A further decrease was noted when CRM genes were pre-induced by CuSO_4_. In addition, higher Cu concentrations lead to a higher proportion of the initial cell population entering the VBNC state (Ordax et al. 2006; Jiang et al. 2016; del Campo et al. 2009; Grey and Steck 2001b). These results corroborate that the Cu ions are inducing the VBNC state and that CRMs play an important role in the Cu-induced VBNC state by preventing the buildup of excessive amounts of Cu in the cytoplasm. Cu detoxification occurs via efflux systems as well as chemical modification of Cu^+^. In Gram-negative bacteria, Cu efflux can be mediated by P_I_-type ATPases (e.g. CopA) and tripartite HME-RND-driven systems (Heavy Metal Efflux Resistance-Nodulation-Division) such as the CusCBA Ag/Cu efflux pump. Periplasmic multicopper oxidases such as CueO and PcoA add another layer of defense by oxidizing Cu^+^ to the less toxic Cu^2+^ (Hobman and Crossman 2015; Bondarczuk and Piotrowska-Seget 2013). These systems are commonly regulated by Cu-responsive transcriptional regulators belonging to at least nine different classes (Rademacher and Masepohl 2012). In Gram-positive bacteria, Cu efflux also seems to be largely mediated by P-type ATPases such as CopA and CopB (Solioz et al. 2010).

While it is clear that CRM activity protects the cell against Cu stress, this protection is not sufficient to maintain culturability under increased Cu concentrations. Culturability can often be regained upon chelation of excess Cu ions by EDTA, DDTC, or complex media such as LB. In the case of resuspension in growth media, we cannot rule out effects other than direct chelation of Cu ions. Conversely, spontaneous resuscitation, without the addition of chelating agents, has been observed in *Cupriavidus*, *Ralstonia*, *Agrobacterium*, and *Rhizobium* (see Table 1). This behavior occurs only upon low Cu toxicity. In *Cupriavidus*, spontaneous resuscitation was only observed in strains containing the pMOL30 megaplasmid, highlighting the necessity of CRM in this process. While it would be interesting to further compare the extent of VBNC state induction to the activity and complexity of CRM in the strains listed in Table 1, the multiplicity of the experimental conditions largely prohibits this analysis. Indeed, retesting these strains in more standardized conditions could provide essential results in this regard. While the role of CRM in (spontaneous) resuscitation requires further study, the risk for VBNC cells escaping common detection strategies while retaining the potential for resuscitation has been emphasized previously (Ding et al. 2017). An additional risk factor is the spread of metal resistance genes within and between bacterial populations repeatedly exposed to metal stress, which could exacerbate this effect. While the Cu-induced VBNC state has been described in (opportunistic) pathogens such as *E. coli* O104:H4 and *P. aeruginosa* PAO1, no *in vivo* studies have been undertaken to determine the generation of difficult-to-detect VBNC cells by existing Cu-based therapies. However, such studies could provide interesting and relevant data for the medical field.

## Concluding remarks

**Fig. 1:**
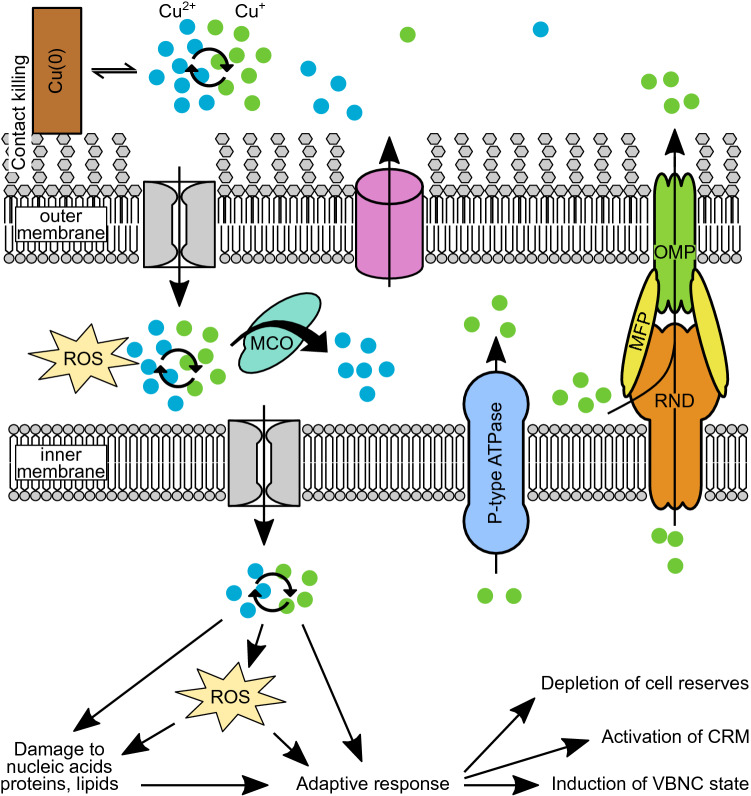
Overview of pathways from Cu exposure to induction of the VBNC state

Recent years have seen the curious observation of a Cu-induced VBNC state in many phylogenetically distinct bacterial species. Here we summarized these observations and investigated the mechanisms of VBNC state induction by Cu toxicity (Fig. 1). While more research into this matter is needed, we argue that the VBNC state induced by Cu is the result of an adaptive response to this stressor. Sub-lethal Cu concentrations bring about cellular damage, rendering the cell unable to multiply. In response it will redirect its metabolism to enable reparation of sustained damage and synthesis of CRM. This response is likely under the control of alternative sigma factors. In this sense, the Cu-induced VBNC state is a programmed phenomenon. However, many different stresses can result in non-culturability, so VBNC states seem to be a common consequence of stress rather than a programmed behavior with a single and discrete set of regulators. This is evident from the observation that different stressors capable of inducing a VBNC state, such as starvation, hypochlorous acid, low temperatures and Cu, prompt the bacterial cell to activate resistance mechanisms to each of these stressors separately (Pinto et al. 2015). Thus, it seems counterintuitive to search for common regulators controlling the VBNC state induced by distinct stressors with distinct mechanisms of toxicity. Rather, we could view the VBNC state as the phenotypical result of a damaged cell opting for survival over multiplication, governed by stressor-specific regulatory mechanisms. Whatever the case, it is certain that understanding the particularities of the VBNC state is a crucial task in ensuring satisfactory biosafety and biocontrol. Several uncertainties remain at many levels, from the initial interaction of the cell with Cu ions over the type, site, and extent of damage accrued, to the molecular mechanisms governing the cellular response. Since, in most cases, only a certain fraction of the initial population can be converted to and resuscitated from the VBNC state, one research question in particular concerns the cellular heterogeneity of these processes. In this sense, single cell-oriented techniques may provide much-needed information. All in all, it is clear that these studies would benefit from a standardized multifaceted experimental approach, integrating whole-cell analyses of copper chemistry, single cell proteomics and metabolomics in order to pinpoint the metabolic status of VBNC cells and the sensory system facilitating resuscitation.
